# Generating Recombinant Antibodies against Putative Biomarkers of Retinal Injury

**DOI:** 10.1371/journal.pone.0124492

**Published:** 2015-04-22

**Authors:** Michael R. Kierny, Thomas D. Cunningham, Rachida A. Bouhenni, Deepak P. Edward, Brian K. Kay

**Affiliations:** Department of Biological Sciences, University of Illinois at Chicago (UIC), 845 W. Taylor St, 3240 SES—MC 066, Chicago, IL 60607–7060, United States of America; Naval Research Laboratory, UNITED STATES

## Abstract

Candidate biomarkers, indicative of disease or injury, are beginning to overwhelm the process of validation through immunological means. Recombinant antibodies developed through phage-display offer an alternative means of generating monoclonal antibodies faster than traditional immunization of animals. Peptide segments of putative biomarkers of laser induced injury in the rabbit, discovered through mass spectrometry, were used as targets for a selection against a library of phage-displayed human single-chain variable fragment (scFv) antibodies. Highly specific antibodies were isolated to four of these unique peptide sequences. One antibody against the retinal protein, Guanine Nucleotide-Binding Protein Beta 5 (GBB5), had a dissociation constant ~300 nM and recognized the full-length endogenous protein in retinal homogenates of three different animal species by western blot. Alanine scanning of the peptide target identified three charged and one hydrophobic amino acid as the critical binding residues for two different scFvs. To enhance the utility of the reagent, one scFv was dimerized through a Fragment-crystallizable hinge region (i.e., Fc) and expressed in HEK-293 cells. This dimeric reagent yielded a 25-fold lower detection limit in western blots.

## Introduction

The discovery of biomarkers that are indicative of injury or disease and their sensitive detection is the future of preventive medicine. Biomarkers are biological molecules released by cells into the serum or surrounding fluid in response to a biological state [1]. Detection of certain biomarker proteins that are associated with a condition and are at abnormal concentrations, can aid in prevention, diagnosis, and regression monitoring. Although biomarkers can be of any biological composition, the proteome has the greatest potential for insight into the diseased state of a patient. However, recognizing specific proteins at low concentrations can be challenging when thousands of different proteins are present in a complex sample [2].

Currently there are many biomarkers used routinely for diagnostics. Some examples of injury biomarkers include Neutrophil Gelatinase Associated Lipocalin (NGAL) for acute kidney injury [3, 4], cardiac Troponin I (cTnI) for myocardial infarction [5], and a panel of biomarkers including α-Spectrin II Breakdown products for traumatic brain injury [6]. For diseases, biomarkers of cancers are important since early diagnosis has long been known to improve patient outcome [7]. Some biomarkers for cancer are prostate specific antigen (PSA) for prostate cancer [8, 9], CA 125 for Ovarian cancer [10], and Carcinoembryonic Antigen (CEA) for colorectal cancer [11].

Traditional antibodies, made by animal immunizations and hybridoma immortalization [12], have been excellent tools for identifying enormous numbers of medically relevant proteins; however, there are not nearly enough to cover the proteome, many of the antibodies that are available are not specific [13], they take several months’ time to generate [14], and they are not always renewable. Therefore, to continue to advance preventative medicine and quality of life, newer technologies must be employed to meet the rising need for custom antibodies of newly discovered biomarkers.

Recombinant affinity reagents, developed through technologies like phage-display, provide an alternative route for generating diagnostics for biomarkers [15]. This technology allows for libraries of antibody fragments to be co-expressed with the M13 bacteriophage coat protein III during phage assembly, where they are available to bind an antigen of interest [16]. After an affinity selection procedure, which increases in stringency through three rounds of antibody-antigen incubation, washing, and amplification of the tightest binding sequences, the DNA sequence encoding the selected antibody can be recovered. One significant feature of this technology is the linking of the genotype with the phenotype, where the DNA for the displayed antibody is encapsulated within the phage particle [17].

**Fig 1:**
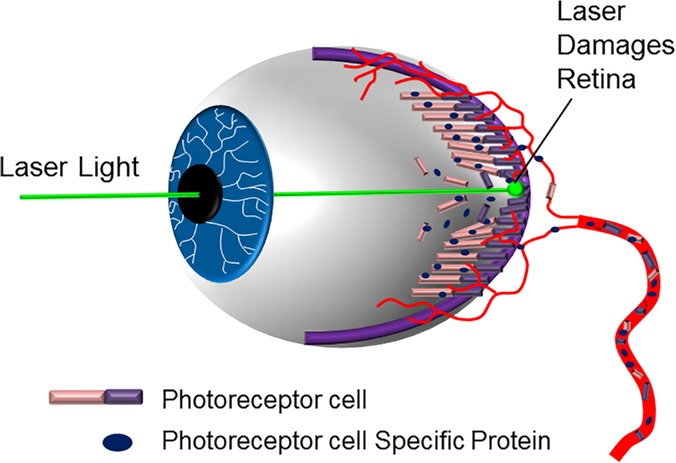
Diagram of retinal injury from laser exposure. As the laser enters the pupil, it is focused by the lens onto the retina. Depending on the intensity and duration of exposure, the cellular layers of the retina can be damaged. The damaged cells and components are cleared from the area of injury and enter the serum where they can be detected as biomarkers and correlated with the laser exposure.

Laser illuminations of commercial and military aircraft pose a serious threat to a pilot’s vision and the safety of the passengers on board. Such events most often occur near airports in cities, where the human population is densest, affordable laser pointers are available, and aircraft maneuver at low altitude. This is also the moment when a pilot is performing the most complex operational procedures that require the greatest concentration and visual acuity [18]. When the laser enters through the pupil (**Fig 1**), the beam is focused onto the retina up to 100,000 times [19] and causes damage by thermal and mechanical means [20]. Nearly 4,000 unauthorized laser illumination events were reported in 2013 [21], which can cause temporary flash-blindness, afterimage, distraction, or severe retinal burns [22, 23]. The degree of retinal damage and the effects to the pilot’s ability to fly the aircraft can vary by situation. Currently, pupil dilation with an exam by an ophthalmologist is the only way to determine the extent of the damage [24], but leaves the pilot unable to fly for 4–8 hours. A low-invasive diagnostic, probing for biomarkers in serum or tears of an exposed pilot to confirm the degree of damage, would be invaluable to the aviation industry.

In a rabbit model of laser induced retinal injury, previously described by the authors (RB and DE) [25], retinal proteins (Guanine Nucleotide-Binding Protein Beta 5 (GBB5), Calcium Channel Voltage-Dependent, L-type, Alpha 1 subunit (CACNA1F), Cyclic Nucleotide Gated Channel Alpha 3 (CNGA3), and Regulator of G-protein Signaling 9 (RGS9), were detected in serum by liquid-chromatography, tandem mass spectrometry (LC-MS/MS) at 4 and 24 hours after retinal exposure to a double frequency Nd: YAG 532 nm laser that created mild or moderate retinal injury (unpublished data). Commercial antibodies were available for these proteins however; in our hands, they did not work in western blot, as is a common occurrence [26]. Further, they were not raised against the same epitopes identified by MS, or the binding region was not disclosed.

Here, we have generated single-chain variable fragment antibodies against four peptides derived from putative biomarkers of laser induced retinal injury, using phage-display. One scFv antibody, against the retinal protein Guanine Nucleotide-Binding Protein Beta 5 (GBB5), was carried further to demonstrate a method to characterize antibodies generated from peptide fragments identified by Mass Spectrometry of serum samples. The scFv was shown to have a ~300 nM affinity and could recognize the full-length, endogenous protein in a western blot. To increase the usefulness of the reagent, the scFv was homodimerized, through genetic fusion with the Fragment crystallizable hinge region (i.e., Fc) of an immunoglobulin G (IgG) isotype molecule, and expressed in Human embryonic kidney 293 (HEK-293) cells. The resulting recombinant scFv-Fc protein gave a 25-fold lower detection limit in western blot and a 4.5-fold improvement in enzyme linked immunosorbent assay (ELISA) signal.

## Materials and Methods

### Ethics Statement

All procedures involving experimental animals were conducted in accordance with the policies of the Animal Care Committee at Northeastern Ohio Medical University; Approved protocol Number 10–039 with following the Statement for the Use of Animals in Ophthalmic and Vision Research adopted by the Association for Research in Vision and Ophthalmology. General anesthesia was carried out with subcutaneous injection of dexmedetomidine (approximately 0.25–0.5 mg/kg) and ketamine (15 −20 mg/kg). An additional injection (1/4 to 1/3 of original dose) after 30 to 45 min was used, if needed, in order to maintain anesthetic state until the collection was complete. Topical: proparacaine HCL 0.5% eye drops was used prior to laser treatment. Atipamazole was used as a reversing agent. Euthanasia was performed under pentobarbital-containing solution. Dutch Belted rabbits were used for the experiments (obtained from Myrtle's Rabbitry Inc., Thompsons Station, TN). All experiments conducting laser exposure to rabbit retina, collection of bodily fluids, as well as methods for anesthesia and euthanasia, are stated in protocol number 10–039 approved by the Animal Care Committee at Northeastern Ohio Medical University. The activity, appetite, eye condition (color of conjunctiva, presence of discharge) and behavior including evidence of ocular discomfort (such as eye rubbing) in rabbits was checked the next day and then at the point of the next body fluid collection. Any animal showing signs of illness were monitored at least once daily until satisfactory resolution of the problem. Laser treatment to the retina is not painful, and does not need pain control in humans after treatment (treatment in humans is done under topical anesthesia). In the unlikely event that evidence of ocular (or other) discomfort was noted, such as blepharospasm, then an analgesic and or other treatment as determined in consultation with the attending veterinarian was administered.

### Peptide Synthesis

Putative rabbit biomarker peptides were identified through LC-MS/MS(n = 27 laser treated and n = 27 mock control, 3 rabbits were used for each laser grade, MVL, GII or GIII, at each time point 1 hr, 4 hr and 24 hr), using published methods [25]. Peptides were synthesized by the protein core facility of the Research Resources Center at the University of Illinois-Chicago. The N-terminus contains a biotin molecule followed by a four amino acid linker composed of Glycine-Serine-Glycine-Serine. The identified peptide of 9–14 amino acids follows the linker and ends with a C-terminal amidation. Peptides were dissolved into sterile phosphate buffered saline (PBS: 137 mM NaCl, 3 mM KCl, 8 mM Na_2_HPO_4_, 1.5 mM KH_2_PO_4_) and stored at -20°C. The peptides synthesized are named after the full-length biomarker they represent: Calcium Channel Voltage-Dependent, L-type, Alpha 1 subunit: CACNA1F#1 (IRWFSHSTR) and CACNA1F#3 (TEGNLEQANQELRIVIK); Cyclic Nucleotide Gated Channel Alpha 3: CNGA3 (RLTRLESQMNRRCCGFSPDRE); Guanine Nucleotide-Binding Protein Beta 5: GBB5 (KLHDVELHQVAERV); and Regulator of G-protein Signaling 9: RGS9 (KLVEVPTKMRV).

### Affinity Selection of the Phage-displayed scFv Library

The scFv phage-display library was a gift from Dr. Mark Sullivan (University of Rochester, Rochester, NY) [27]. The library had been constructed from amplified cDNA of human B-cells with an estimated diversity of 1x10^9^ clones. The library vector contained an N-terminal FLAG tag, followed by the V_L_ chain, a 14 amino acid linker EGKSSGSGSESKAS, the V_H_ chain, and the pIII phage coat protein. The wells of a Nunc Maxisorp 96-well microtiter plate (Nunc) were coated with 50 ng NeutrAvidin (Thermo Fisher Scientific) in PBS and incubated at 4°C overnight. The plate was blocked with 1% casein (Thermo Fisher Scientific) for 1 h and then washed 3 times with PBS with 0.5% Tween 20 (PBST). The scFv phage-display library (with titer of 10^12^ phage/mL) was added in 50 μL volumes to four blocked wells and incubated for 1 h to deselect casein and NeutrAvidin binders. The unbound phage particles were transferred to wells containing 50 ng of biotinylated-peptide and incubated for 2 h at room temperature with shaking. The wells were washed 5 times with PBST. The bound virions were recovered using 50 μL of 100 mM Glycine-HCl, pH 2.0 for 10 min. The pH of the solution, which contained the eluted phage particles, was neutralized with 3 μL of 2 M Tris (pH 10), and was used to infect 750 μL of TG-1 *Escherichia coli at* mid-log phase. After 1 h incubation at 37°C, without shaking, the cells were spread on a Luria Bertani broth (LB) agar plate (10 g/L tryptone, 5 g/L yeast extract, 10 g/L NaCl, 15 g/L Agar), containing 50 μg/mL Carbenicillin (Cb), and incubated overnight at 30°C.

The following day, the lawn of bacterial colonies was scraped into 15 mL of LB/Cb. To 40 mL of LB/Cb was added 100 μL of the scraped cells, and the culture grown to mid-log phase at 37°C (250 rpm shaking). One mL of cells were removed, and infected with M13K07 helper phage (New England BioLabs), at a multiplicity of infection (MOI) of 10, for 1 h at 37°C, with 150 rpm shaking. This mixture was added to 30 mL of LB/Cb/Kanamycin (Kan, 50 μg/mL), and grown overnight at 30°C, with 250 rpm shaking, to allow for virion production.

To precipitate the secreted virions for the second round of affinity selection, the overnight culture was spun down and ~30 mL supernatant adjusted to a final concentration of 500 mM NaCl, 4% PEG_8000_, and incubated on ice for 1 h. This tube was spun at 12,000 rpm for 15 min, and the precipitated virions suspended in 1 mL of 0.5% casein in PBS. This sample was the used for the second round of affinity selection. Rounds #2 and #3 of affinity selection were performed the same as round #1, except that the target was now reduced to 5 ng in a single well, and the PBST washes were increased to 7 times, with harsh vigorous pipetting up and down in between.

After the final round of infection and plating of clones, 94 colonies were used to inoculate wells of a 96-well deep plate containing 100 μL of LB/Cb and grown to mid-log phase (OD_600_ = 0.4). Two wells contained positive and negative controls of the phage ELISA. To all wells, 200 μL of M13K07 helper phage in LB/Cb and were added without shaking for 1 h at 37°C. Plates were spun at 4,000 rpm, supernatant discarded, and 400 μL of LB/Cb/Kan added for overnight phage expression at 30°C with 250 rpm shaking.

### Phage ELISA

Two 96-well microtiter plates were coated overnight with 50 ng/well NeutrAvidin in PBS. The wells were blocked with 1% casein in PBS for 1 h. To one plate was added the target biotinylated peptide at 50 ng/well and incubated for 1 h. After washes in PBS and PBST, 50 μL of supernatant from each well of the overnight culture of expressed phage (1x10^12^ phage/mL) was added to the corresponding well of the coated target or background no-target plate, and incubated for 1 h. Washes of PBST and PBS followed. For detection of the bound phage particles, 50 μL of anti-M13-Horse Radish Peroxidase (HRP; GE Healthcare), diluted to 1:5,000 in PBS, was added to all wells. Following washes of PBST and PBS, 50 μL of 2,2'-azino-bis (3-ethylbenzothiazoline-6-sulphonic acid (ABTS; Sigma-Aldrich) in 50 mM Sodium Citrate (pH 4), with 0.03% H_2_O_2_, was added, and color change recorded at absorbance wavelength 405 nm using a FLUOstar OPTIMA (BMG Labtech) spectrophotometer. Comparing the absorbance of corresponding wells on background and target plates identified specific binders.

### Cloning and bacterial expression

The positive clones were grown in cultures overnight and the plasmid DNA was prepared using a Wizard MiniPrep DNA purification column (Promega). The scFv regions were sequenced using a forward primer, CTGTCATAAAGTTGTCACGGCCGA, and reverse primer, CCCCTTATTAGCGTTTGCCATCTT. Unique clones were then subcloned, using *HindIII* and *SalI* restriction endonucleases (New England BioLabs) to insert the DNA into the expression plasmid, pKP300ΔIIIΔAP [28], with an in-frame N-terminal Flag epitope tag, C-terminal (His)_6_ tag, and an OmpA signal sequence targeting the scFv to the periplasm. Expression is performed in autoclaved, low phosphate media [29], which consists of 3.57 g ammonium sulfate, 0.71 g sodium citrate dihydrate, 1.07 g potassium chloride, 5.36 g Yeast Extract, 5.36 g Hycase SF Casein hydrolysate, (pH adjusted with potassium hydroxide to 7.3), 7 mL 1 M magnesium sulfate and 14 mL 1 M glucose. The culture is incubated with 50 μg/mL Cb overnight at 30°C (250 rpm shaking).

For small-scale expression, the infected bacterial cells were spun down and resuspended in a lysis solution consisting of Bugbuster in PBS (Novagen) and Benzonase Nuclease HC (Novagen) for 20 min. After pelleting the cell debris, the scFv was purified from the supernatant by immobilized metal affinity chromatography (IMAC), using His·Mag Agarose Magnetic beads (Novagen) and a Kingfisher mL robot (Thermo Fisher Scientific). Antibodies were eluted into 500 mM Imidazole, 500 mM NaCl, and 20 μM Tris-HCl (EB), and stored at 4°C, or at -20°C in 15% glycerol.

For large scale expression, infected cells were added to 200 mL of low phosphate media, 50 μg/mL Cb, and allowed to grow 22–24 h at 30°C with 250 rpm shaking. Cultures were spun down and prepared for sonication by resuspending the cell pellet in 25 mL of filter sterilized equilibration buffer (50 mM sodium phosphate, 300 mM sodium chloride, pH 7.4) on ice. The complete EDTA free protease Inhibitor cocktail (Roche Applied Science) was added and performed sonication on ice with 10 sec on sonication, 10 sec off for a total of 10 min, with 50% amplitude using a Sonic Dismembrator (Branson, Model 500). The lysate was spun at 15,000 rpm for 15 min, and the supernatant transferred to a 50 mL centrifuge tube. Agarose was prepared by washing 200–300 μL of Clontech His-60 Ni Superflow resin (60 mg/mL binding capacity, Clontech Laboratories) twice with equilibration buffer. The washed resin was added to the cleared lysate and incubated at 4°C for 2 h, with tumbling. The lysate was spun down for 2 min at 1,000 rpm in a microcentrifuge; the supernatant removed and resuspended in 1 mL wash buffer, containing 50 mM sodium phosphate, 300 mM sodium chloride, 10 mM Imidazole, pH 7.4. The sample was recentrifuged and the wash buffer removed as above. Washes were repeated three more times. The protein was eluted in 250 μL filter-sterilized Elution buffer (50 mM sodium phosphate, 300 mM sodium chloride, pH 7.4, 300 mM Imidazole) for 10 min. The protein concentration was determined with a NanoDrop A280 spectrophotometer (Thermo Fisher Scientific) and purity assessed by sodium dodecyl sulfate-polyacrylamide gel electrophoresis (SDS-PAGE).

### Soluble scFv ELISA

Microtiter plate wells were coated with 50 ng/well NeutrAvidin or BSA, overnight at 4°C. The wells were washed with PBST, and 50 μL of 50 ng/well biotinylated peptide was added and incubated for 1 h. After washing, 200 μL of 5% Non-Fat Dried Milk in PBST was added to block the wells. After 1 h, the wells were washed with PBST and PBS, and 50 μL of 10 μg/mL purified scFv antibody in PBS were added and incubated for 1 h, with shaking. Wells were washed with PBST, and 50 μL of ABTS in 50 mM sodium citrate (pH 4), 0.03% hydrogen peroxide was added to all wells. The color change was recorded at 405 nm with a microtiter plate reader.

### Western blot of retinal lysates

Retinal lysates from rabbit (*Oryctolagus cuniculus*) and mouse (*Mus musculus*) were harvested according to approved IACUC protocol. Chicken (*Gallus gallus*) eyes were purchased from a local Amish butcher (Alliance Poultry Farms, Chicago, IL). Retinas and whole eyes were homogenized in 10 mM Tris-HCl, pH 7.4, 1 mM EDTA, and 200 mM Sucrose. Lysate was spun down and 5 μL of the supernatant used for SDS-PAGE and western blotting. Protein samples were resolved in Tris-Glycine-SDS buffer with a 12% precast Mini-Protean TGX polyacrylamide gel (Bio-Rad), which was electrophoresed at 12 mA for 1.5 h. The protein samples were transferred overnight to a polyvinylidene fluoride (PVDF) membrane (Millipore), at 25 V in Tris-Glycine-SDS buffer with 20% Methanol. Non-specific binding to the membrane was blocked with 5% Non-Fat Dried Milk in PBST for 1 h. After five min washes with PBST and PBS, anti-GBB5-H9 scFv was added at a concentration of 1 μg/mL in 25 mL PBS and incubated with shaking for 2 h. The blot was then washed for five min in PBST and then PBS, before adding the secondary antibody, anti-Flag-HRP M2 (Sigma-Aldrich), which was diluted 1:5000 in PBS. After 1 h incubation, with shaking, the blots were washed five minutes with PBST and then with PBS. Detection of immune complexes was performed using the Enhanced Chemiluminescence (ECL) Prime reagent (GE Healthcare) and imaged using a Storm 860 Phosphorimager (Molecular Dynamics).

### Alanine scanning of GBB5 peptide

Thirteen versions of the GBB5 peptide were synthesized, with alanine replacing each consecutive position, one at a time, in the peptide. The N-terminus contains a biotin molecule followed by a four amino acid linker composed of Gly-Ser-Gly-Ser. For the ELISA, 250 ng of each peptide was immobilized in triplicate on a NeutrAvidin coated Nunc 96-well Maxisorp plate. (The assay was conducted similar to the ELISA described above.) After blocking with 1% casein (Thermo Fisher Scientific), 1 μg/mL of anti-GBB5-H9 scFv or anti-GBB5-A1 scFv protein was added in PBST for 1 h. The secondary antibody, anti-FLAG-HRP, was added at 1:5,000 dilution in PBST and incubated for 1 h. The signal was detected with ABTS and hydrogen peroxide, and scanned at 405 nm in a microtiter plate reader.

### ScFv-Fc Expression

The plasmid pBIOCAM5 [30], containing the Fragment crystallizable (Fc) and hinge region of the human IgG, was a gift from Dr. John McCafferty (University of Cambridge, UK). This construct contained a CMV promoter for expression in HEK-293 Freestyle cells, the constant C_H2_-C_H3_ regions of the human Fc, followed by a (His)_6_ tag and a C-terminal tri-FLAG epitope tag. The coding region for the H9 scFv was subcloned upstream of the Fc through the *NcoI* and *NotI* restriction sites. The Fc forms homodimers through disulfide bond formation between cysteine residues.

Using standard cell culturing techniques, HEK-293 F’ (Invitrogen) cells were grown as a 50 mL suspension in Freestyle 293 serum-free media (Gibco) to a density of 1x10^6^ cells/mL at 37°C, in 10% CO_2_, with 140 rpm rotation. After two passages, the cells were transiently transfected using 20 μg of 0.2 um filter sterilized pBIOCAM5 DNA and 50 μL of 1 mg/mL Polyethylenimine 25 kDa (Polysciences Inc.), vortexed, and incubated for 10 min at room temperature. Cells were returned to the 250 mL flask and shaken for seven days. The cells were harvested and the supernatant recovered. cOmplete EDTA-free Protease Inhibitor Cocktail (Roche Applied Science) was added and the scFv-Fc antibodies purified in batch with Clontech His-60 Ni Superflow resin, as described above. The scFv-Fc fusion was eluted in filter-sterilized Elution buffer [50 mM sodium phosphate, 300 mM sodium chloride (pH 7.4), 300 mM Imidazole] and stored at 4°C.

### Generation of peptide-MBP fusion control

Primers were designed to include the DNA sequence of the peptide during the PCR amplification of the maltose binding protein (MBP) in the pAT224 vector (gift of Dr. Andreas Plückthun, University of Zurich; GenBank #AY327139). The forward oligonucleotide was synthesized (Integrated DNA Technologies) to contain (from 5' → 3') the *NcoI* restriction endonuclease recognition site, the *E*. *coli* codon optimized sequence encoding the biomarker peptide, and 20 bases of complementary sequence to allow hybridization to the vector and PCR priming. Forward primers included 8 additional bases on the 5' end to allow for restriction enzyme digest of the amplicon. The sequences of the primers are (from 5'→3'): GBB5fwd: (ATTATATTCCATGGCCAAACTGCATGATGTGGAACTGCATCAGGTGGCGGAACGCGTGGGGAAAACTGAAGAAGGTAAACTGGT); RGS9fwd: (ATTATATTCCATGGCCAAACTGGTGGAAGTGCCGACCAAAATGCGCGTGGGGAAAACTGAAGAAGGTAAACTGGT). The reverse primer, CGTTCTGAACAAATCCAGATGGAGT, anneals downstream of the 3' end of the MBP sequence and the amplicon includes the *HindIII* site for subcloning purposes.

The reaction was performed with 0.3 μM of the forward and reverse primers, 300 ng pAT224-MBP template, 1.25 U of AccuPrime Pfx DNA Polymerase (Invitrogen), supplied buffer (includes dNTPs), in 25 μL total volume. Thermal cycling is as follows: denature 2 min at 95°C, denature 15 sec at 95°C, anneal 30 sec at 55°C, elongate 85 sec at 68°C, cycle back to step 2 for 29 more times, and final elongation at 68°C for 5 min. The reaction was purified with a cleanup kit (QIAquick, Qiagen) and both the polymerase chain reaction (PCR) amplicon and pAT224-MBP were digested with *NcoI* and *HindIII* restriction endonucleases (New England BioLabs). After gel purification (QIAquick, Qiagen) the vector and amplicons were ligated at a 1:9 ratio (vector:insert) using 100 U of T4 DNA Ligase (New England BioLabs) in 20 μL total volume and incubated at 16°C overnight. The reactions were spot dialyzed and electroporated into electrocompetent XL-1 Blue cells and plated on LB/Cb overnight at 30°C.

Upon sequence confirmation, the recombinants were grown to an optical density (OD) of 0.7 at 600 nm wavelength, and induced with 500 μM isopropyl-β-D-thiogalactopyranoside (IPTG) for 6 h at 37°C. Cells were pelleted and freeze-thawed at -80°C before sonication. The cleared lysate was batch incubated with nickel-nitriloacetic acid (Ni-NTA) resin (Qiagen) for 5 h at 4°C. Protein was purified using washes of PBS + 10 mM imidazole and elution into 500 mM imidazole, 20 mM Tris-HCl, 500 mM NaCl. Fractions were stored with 30% glycerol at -20°C. The expected size of the recombinant protein is 46.2 kilodaltons (kDa).

### Comparison of scFv with scFv-Fc

Two sets of the GBB5-MBP protein was resolved at decreasing concentrations (2 to 0.01 μg) in a 12% precast Mini-Protean TGX polyacrylamide gel (Bio-Rad) in Tris-Glycine-SDS buffer, at 12 mA for 1.5 h. The protein was then transferred to polyvinylidene fluoride (PVDF) membrane (Millipore) overnight (12 h) at 25 V in Tris-Glycine-SDS buffer with 20% Methanol. The blots were split in half and identically treated until addition of the antibody. The non-specific protein binding sites on the membranes were blocked with 5% Non-Fat Dried Milk in PBST for 1 h. Afterward, the blots were washed once with PBST and once with PBS, for 5 min each. Thirty nM of anti-GBB5-H9 scFv and 10 nM of anti-GBB5-H9 scFv-Fc version was added separately in 25 mL volumes of PBS, and incubated with the blots, shaking for 2 h. Blots were washed once with PBST and PBS for 5 min each. The secondary antibody, anti-FLAG-HRP M2 (Sigma-Aldrich), was diluted 1:5,000 in PBS, added to the blot, and incubated for 1 h, while shaking. The blots were washed once with PBST and PBS for 5 min each. Detection was performed using ECL Prime reagent (GE) and imaged using a Storm 860 Phosphorimager (Molecular Dynamics).

The ELISA was performed as mentioned earlier. Briefly, 50 μL of 15 nM NeutrAvidin was coated on 96-well Maxisorp microtiter plates overnight at 4°C. Wells were blocked with 1% casein in PBS. The peptides were immobilized in 50 μL of PBST at 25 nM. The scFv was added in 50 μL volumes of PBST at 30 nM and the Fc was added at 10 nM. Recombinant antibodies were detected with anti-FLAG-HRP, developed with ABTS, and the absorbance of wells measured at 405 nm.

### Affinity estimate by photonic crystal biosensing

The SRU Biosystems BIND Explorer photonic crystal biosensor was used to detect real-time and label-free binding of scFv antibody to the target peptide [31]. The software program, Experiment Management System (EMS) V2.1 was used to record the data. Fifty ng/well of peptide in PBS was immobilized onto Streptavidin coated 96-well plate-based photonic biosensors and the baseline was set after 5 min. The wells were then washed with PBST, PBS, and blocked with filtered 5% BSA in PBS, for 1 h. After washing with PBST and PBS, the molecular interactions were allowed to reach equilibrium in 50 μL of elution buffer (EB) per well. For each scFv antibody, 50 μL of decreasing concentration was added to 7 wells, and the binding responses recorded after traces had reached a plateau. Overnight end-point values of change in peak wavelength were used to draw the response curve. Data was analyzed using either the instrument’s software or OriginPro 8.5 (OriginLab Corp.).

## Results and Discussion

### Phage-display selection on peptide biomarkers

Retinal injury peptides, identified by liquid chromatography and tandem mass spectrometry, were used as targets for recombinant antibody generation. The protein targets chosen were Calcium Channel Voltage-Dependent, L-type, Alpha 1 subunit (CACNA1F), Cyclic Nucleotide Gated Channel Alpha 3 (CNGA3), Guanine Nucleotide-Binding Protein Beta 5 (GBB5), and Regulator of G-protein Signaling 9 (RGS9). These were selected because peptides corresponding to these four proteins were identified in the serum of rabbits that experienced laser induced retinal damage. We decided to generate recombinant antibodies for two reasons. First, the commercially available antibodies for these antigens are of poor quality. Second, because the state of the protein is unknown once released by the retina upon laser damage, and it likely degrades when it circulates through the rabbit's system [32, 33], we decided to generate recombinant antibodies that recognize linear epitopes. Such reagents can be employed in Stable Isotope Standards and Capture by Anti-Peptide Antibodies (SISCAPA) assays [34], a multiple reaction monitoring assay, where digested biomarker proteins are antibody-enriched from a sample before quantitative mass spectrometry.

**Fig 2:**
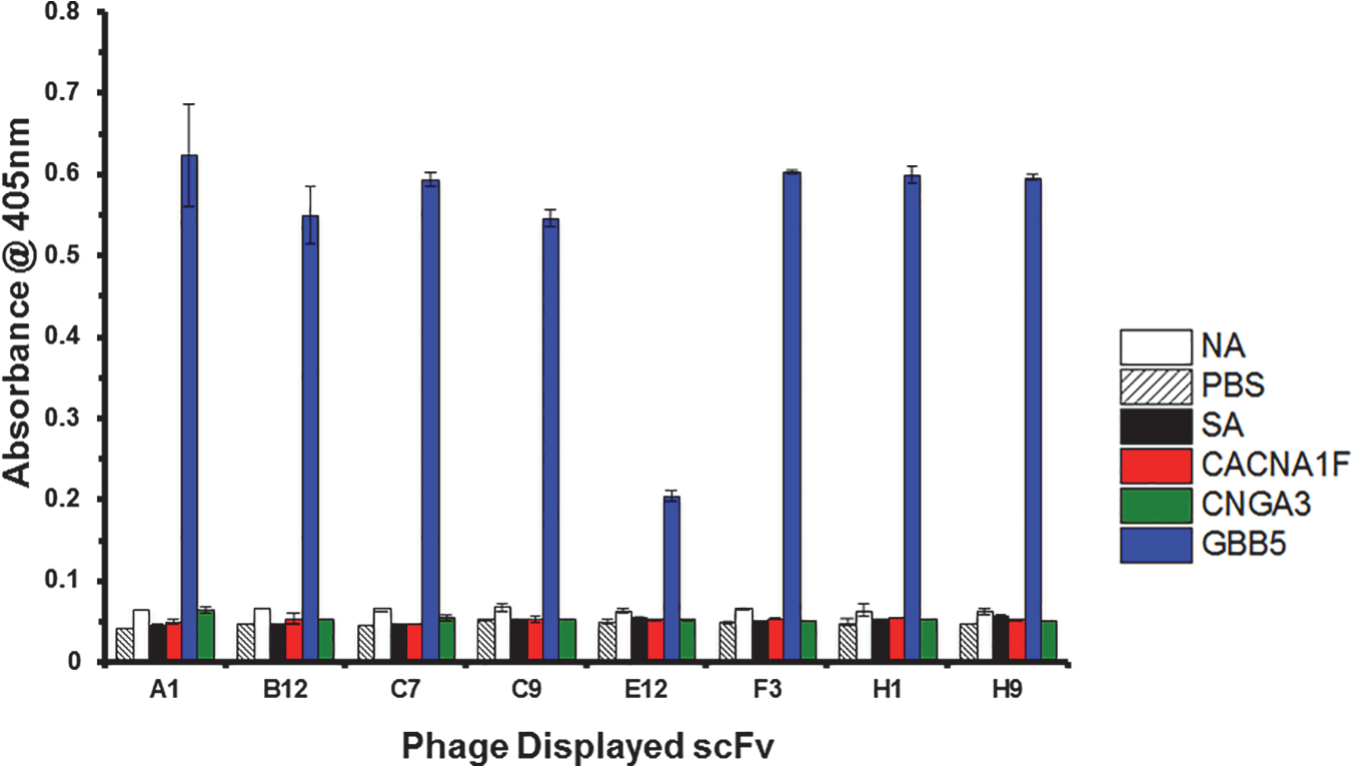
ELISA of scFv-displayed virions against the GBB5 peptide. ScFv antibodies, raised against the GBB5 peptide, are displayed on phage particles and used to probe peptides, which are captured on microtiter plate well surface. The phage-displayed scFvs are named according to the well location of the 96-well screen in which they were originally found. Specificity is determined by the intensity of the absorbance signal for the target peptide (GBB5), compared to the background NeutrAvidin (NA), Streptavidin (SA), Phosphate Buffered Saline (PBS) coated wells or unrelated peptides, CACNA1F and CNGA3.

To generate antibodies against these peptides, we employed a M13 bacteriophage library displaying human V_L_ and V_H_ regions, cloned from B-cells and joined by a 14 amino acid linker region. The library, with a diversity of 1 x 10^9^, was used to affinity select with a biotinylated target peptide immobilized through Streptavidin on a 96-well plate. After three rounds of affinity selection and recovered phage amplification, several scFvs were isolated for each peptide antigen (**Table 1**). Clones with signal intensities ≥ two-fold over background in ELISA were chosen for further characterization. Unique clones were identified by Sanger DNA sequencing. Even though we succeeded in generating anti-peptide scFvs to all four targets, we decided to focus on the GBB5 target. We had generated numerous scFv binders to GBB5 and the crystal structure, in complex with the RGS9 protein (Protein Data Bank entry 2PBI) [35], shows that the peptide sequence, which we used as the selection target for generating a cognate scFv, is exposed on the surface of the protein. Therefore, this epitope might be accessible, even in the intact protein, for interaction with its cognate scFv. As seen in **Fig 2**, the anti-GBB5 scFvs are quite specific.

**Table 1:** Summary of putative biomarker peptides and antibodies generated.

Biomarker	Peptide Target	Organism Match	AAs	Unique Clones
CACNA1F Peptide 1: Calcium Channel Voltage-Dependent, L-type, Alpha 1 subunit	IRWFSHSTR	Human, Rat, Mouse	9	1–4
CACNA1F Peptide 3: Calcium Channel Voltage-Dependent, L-type, Alpha 1 subunit	TEGNLEQANQELRIVIK	Human	17	4
CNGA3: Cyclic Nucleotide Gated Channel Alpha 3	RLTRLESQMNRRCCGFSPDRE	Mouse	21	8
GBB5: Guanine Nucleotide-Binding Protein Beta 5	KLHDVELHQVAERV	Human, Rat, Mouse	14	6
RGS9: Regulator of G-protein Signaling 9	KLVEVPTKMRV	Rat	11	3

The biomarker is the full protein matched to the peptide target that was discovered by MS. The organism match are the animals the peptide is orthologous to in addition to rabbit. The number of clones is defined by scFvs with unique CDRs that bind specifically to their target peptide.

**Fig 3:**
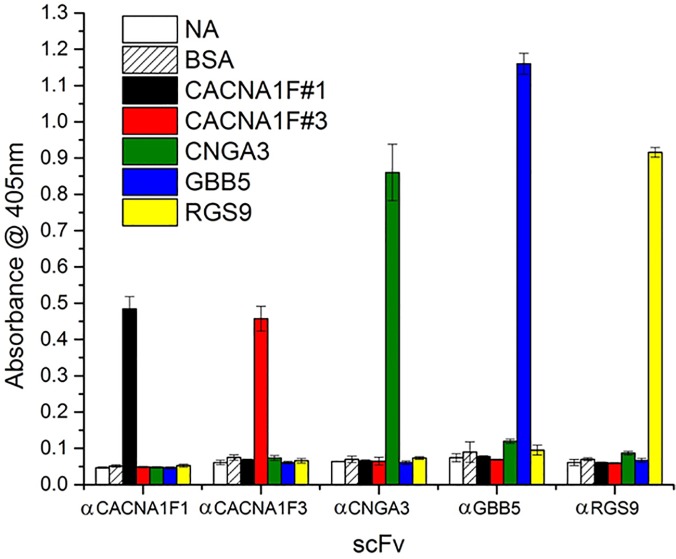
Five scFvs in a soluble ELISA retain their specificity. After transferring the scFv coding region from the phagemid into an expression vector, these five scFvs were expressed in soluble form and purified. To demonstrate the specificity of each antibody, the ELISA was performed against all biomarker peptides available.

Upon subcloning the coding regions of the scFvs into a low-phosphate inducible vector, pKP300ΔIII [28], and electroporation into TG-1 *E*. *coli*, the soluble forms of the overexpressed scFv antibodies were confirmed to bind their peptide targets selectively. As seen in the ELISA of five soluble scFvs selected against five different biomarker peptides, the scFvs give specific signals for their cognate antigens that were 4–10 times over the background (**Fig 3**).

### Affinity estimates using photonic crystal biosensor

**Fig 4:**
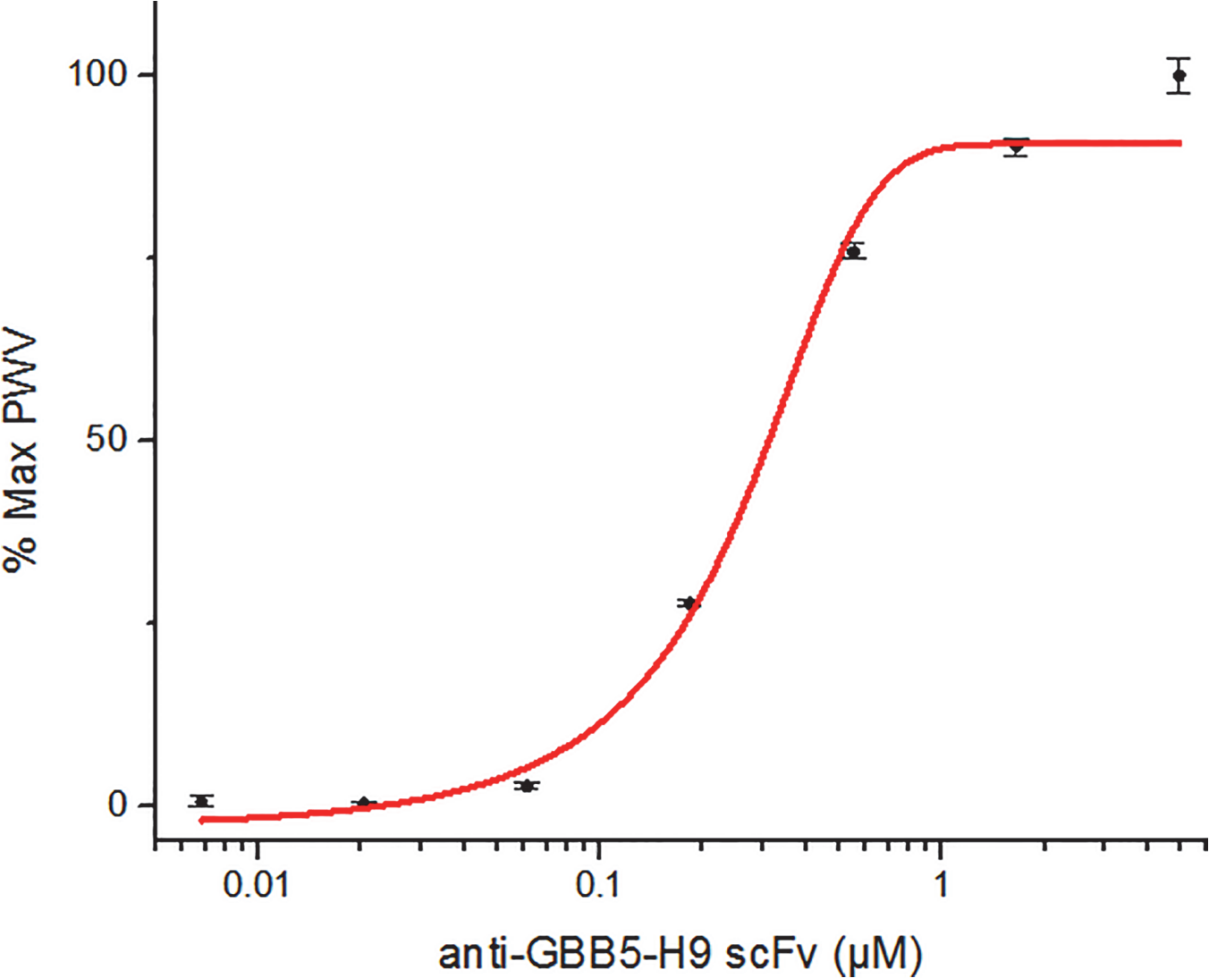
Photonic crystal binding curve. The anti-GBB5-scFv H9 was titrated across wells of a streptavidin-coated, photonic crystal, 96-well, biosensor plate, with the GBB5 peptide immobilized on the well surface. Using the BIND reader, real-time, label-free binding was recoded until the change in peak wavelength plateaued. Plotting the percentage of the max peak wavelength to the log concentration of the scFv, gives a sigmoidal dose response curve. The half maximal effective concentration (EC_50_) of ~ 300 nM was used to estimate affinity.

The photonic crystal binding assay [31] was utilized to estimate the affinities through end-point readings. The biosensor consists of a photonic crystal coated plastic film in a standard 96-well plate. Binding of protein to the surface changes the dielectric permittivity, which results in a shift in the Peak Wavelength of the reflection of an illumination source that is positioned below the plate. The real-time measurements of the instrument allowed for visual identification of equilibrium of the binding interaction, so true end-point values could be used to estimate K_d_. Further, the output signal is stable and will not saturate after a short period of time like the chromographic signal saturation of ELISAs. These measurements were conducted for anti-GBB5 scFvs, which had the highest signal to background ratio in the phage ELISA. Kinetic determinations of K_d_ for anti-GBB5-H9 were estimated by fitting a dose response curve and calculating the half maximal effective concentration (EC_50_) value to be ~300 nM (**Fig 4**). The tightest binding scFv was the anti-GBB5-H9, which had an affinity value about 10-fold greater than the others. Although the differences between the GBB5 binders H9 and A1 is not apparent from the phage ELISA (**Fig 2**), the affinity is 10-fold greater in the H9 and it recognizes the full-length protein in western blot. It is interesting to note that two scFvs with different dissociation constant values can yield comparable phage ELISA results, indicating titrating the antibody, target, or adding a competitor in solution are better at discriminating relative binding strengths than end-point phage ELISA assays.

### Detection of the Cognate protein in Western Blot

**Fig 5:**
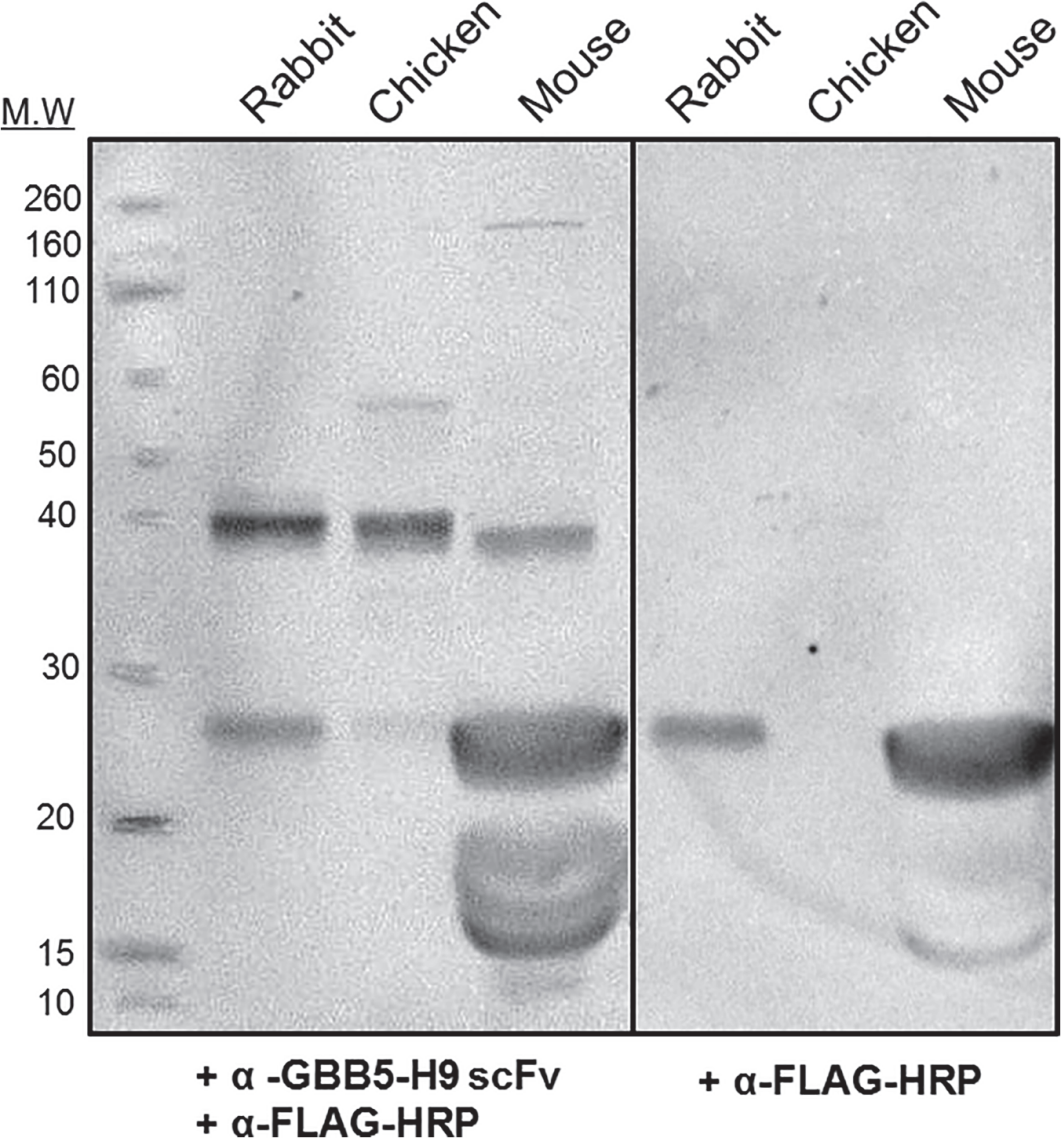
Western blot of retinal lysates. The anti-GBB5-scFv H9 was used to probe retinal lysates from chicken, mouse, and rabbit. The expected size of rabbit and mouse GBB5 is 38.7 kDa, whereas the expected size for the chicken homolog is 38.8 kDa. The left panel is probed with the scFv antibody and the secondary anti-FLAG-HRP antibody. The right panel serves as the negative control (i.e., probed with the secondary antibody only). The total lysates loaded for chicken, mouse, and rabbit are ~35 μg, ~ 50 μg, and ~35 μg, respectively.

To evaluate the ability of the anti-GBB5-H9 scFv to recognize the endogenous antigen, western blotting analysis was performed on retinal lysates. Retinal lysates from chicken (*Gallus gallus*), rabbit (*Oryctolagus cuniculus*), and mouse (*Mus musculus*), were probed with 1 μg/mL of the anti-GBB5-H9-scFv antibody (**Fig 5**). In the chicken, human, mouse, and rabbit genomes, the sequence of the peptide used for affinity selection experiments is identical. The expected molecular weight of the GBB5 protein is 38.7–38.8 kDa for these three organisms. Specific recognition can be seen near the 40 kDa band of the standard protein ladder. (Additional bands can be attributed to non-specific binding of the anti-FLAG-HRP secondary antibody, as seen in the adjacent panel, indicating the scFv is more specific than appears in the left panel) Unfortunately, long wash steps reduced the signal of the scFv to undetectable levels, consistent with the observation made with the photonic crystal biosensor that the scFv has a low affinity.

### Alanine Scanning of anti-GBB5 scFvs

**Fig 6:**
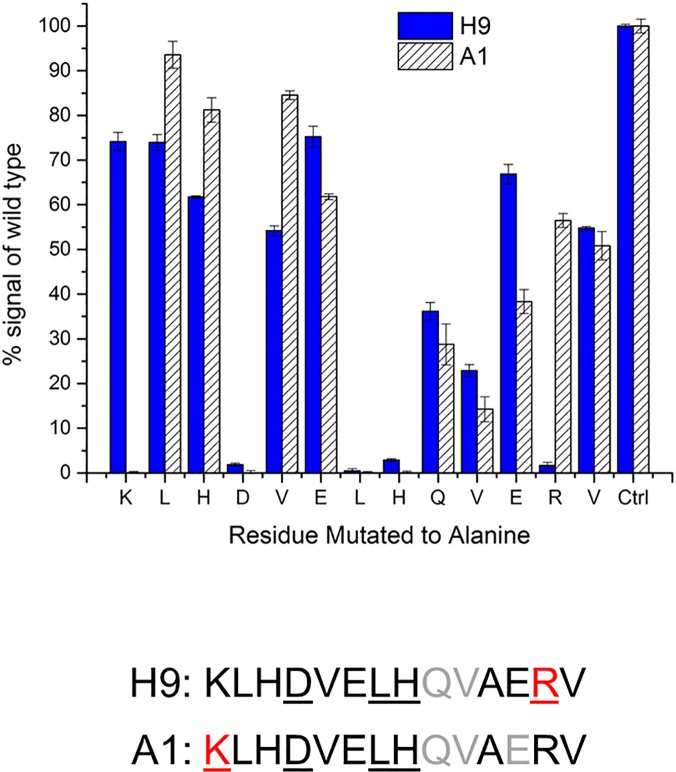
Alanine scanning of important residues. A series of GBB5 peptides were synthesized, with Ala sequentially replacing each residue in the sequence. The position with an alanine already present was left un-changed. Two anti-GBB5 scFvs were used in the ELISA; the binding values for H9 and A1 are 300 nM and 4–6 μM, respectively. Complete loss of signal for an alanine substitution, as compared to the wild-type control (Ctrl), was deemed an important residue (A). The important residues are summarized in (B) as underlined. The red font highlights the different crucial residues for H9 binding, as compared to the A1. Gray font indicates a > 50% reduction in signal when a residue was replaced.

To resolve which residues of the GBB5 peptide antigen are important for the recognition by two different anti-GBB5 scFvs, we ordered a set of 13 peptides, with each amino acid position is replaced one at a time with alanine. After immobilizing biotinylated forms of the peptides in microtiter plate wells coated with streptavidin, we performed ELISAs with soluble scFv protein and compared the signals to the wild-type peptide. We interpreted any decrease in binding due to alanine replacing a residue in the peptide that contributes to recognition by the scFv. Both the H9 and A1 scFvs (**Fig 6A**) interact strongly with the Asp, Leu, and His residues (underlined), as replacement of any one of these residues with an Ala, results in loss of binding. The main difference between the tight binding, nanomolar affinity clone, H9, and the micromolar binding clone, A1, is unique recognition of a single additional residue in the peptide sequence for each (**Fig 6B**). The tight H9 clone interacts strongly with the positively charged Arg on the C-terminus of the peptide (red), whereas A1 requires the positively charged Lys on the N-terminus (red). Unfortunately, the difference in binding strength renders the A1 reagent too weak to be useful in a western blot. Even though both A1 and H9 clones bind the same peptide, the Complementarity Determining Regions (CDRs) of the two scFvs (**S1 Fig**) are only 40% similar, which is not surprising considering the various families of V_H_ and V_L_, which were used for library construction [27].

### Comparison of the binding properties of an scFv with scFv-Fc

**Fig 7:**
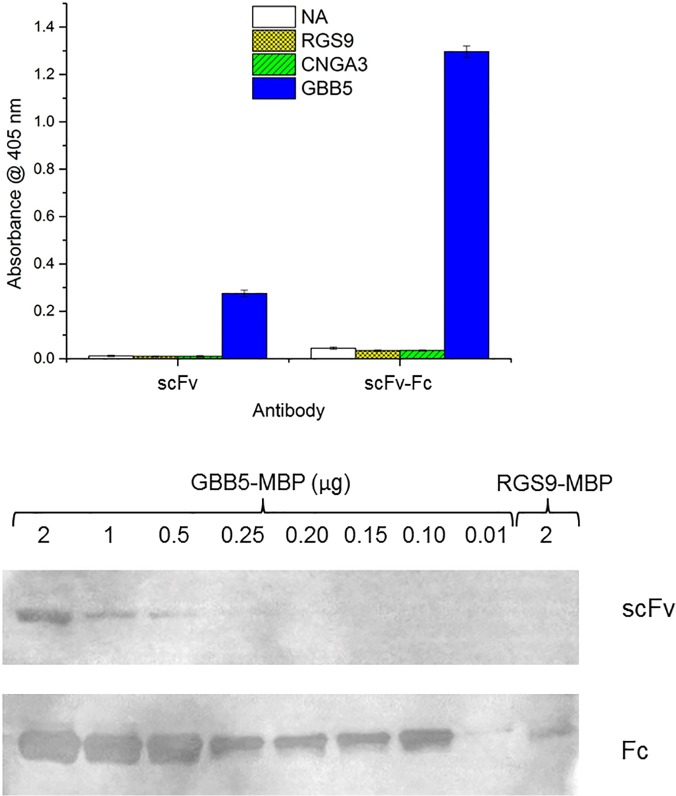
Comparison of scFv and the Fc format of the anti-GBB5-H9. (A) Target peptides are immobilized on a NeutrAvidin-coated microtiter plate at 25 nM and assayed with 30 nM of monomeric *E*. *coli* expressed anti-GBB5 H9 scFv or 10 nM of the HEK-293 expressed bivalent Fc format. The secondary antibody anti-FLAG-HRP is added and subsequent chromogenic reagent. Absorbance is recorded at 405 nm. (B) The GBB5-MBP fusion protein (46 kDa) is detected on PVDF membrane using the anti-GBB5-H9 scFv or Fc format of the H9 clone. Amount loaded is in μg and decreases from left to right. The negative control included is the RGS9-MBP fusion protein at 2μg.

To improve the usefulness of the anti-GBB5-H9 scFv, we decided to dimerize the antibody, and enhancing the apparent affinity of the scFv through avidity [36]. The coding region of the H9 scFv was fused to the C_H2_-C_H3_ region from a human IgG [37]. Subsequent transient transfection and extracellular expression in the human cell line, HEK-293, allowed for fast and easy purification of the scFv-Fc fusion protein from the culture media. Size exclusion chromatography and denaturing SDS-PAGE confirmed that the scFv-Fc was a homodimer, held together in part through disulfide bonds. The signal strength in ELISA of immobilized GBB5 peptide was about 4.5-fold higher for the scFv-Fc compared to the soluble scFv, indicating the apparent affinity had been improved, without altering its specificity, by the dimerization (**Fig 7A**). We then compared the two forms of the H9 scFv in western blots. However, as full-length recombinant GBB5 protein was difficult to express in bacterial and HEK-293 cells (data not shown), we fused the 14 amino acids of the GBB5 peptide in frame to the N-terminus of the *E*. *coli* maltose binding protein (MBP). As seen in **Fig 7B**, the scFv-Fc protein has a limit of detection 25-fold lower (limit of detection, 10 ng) when compared with the scFv (limit of detection, 250 ng) of the MBP-GBB5 peptide fusion in western blots.

In this study, we report using an scFv phage-display library to select for antibodies that bind specifically to short peptides of putative biomarkers of retinal injury. These peptides were implicated as biomarkers in a mass spectrometry analysis of serum from rabbits that were exposed to laser light and incurred retinal damage as a result. One of the recombinant scFv antibodies that were generated against the GBB5 peptide was estimated to have an affinity of 300 nM, and subsequently confirmed to bind the endogenous protein in western blot of retinal lysates from three vertebrate species.

Dissociation constants of 300 nM, were determined to be the upper limit for scFvs to yield a result in western blots. Presumably, antibodies with weaker dissociation constants wash off blots too quickly to generate a detectable signal [38]. For immunoprecipitation experiments to succeed, the affinity likely has to be five to ten times greater than the scFvs generated here [39]. To increase functional affinities, the scFvs were dimerized through fusion to a human IgG Fc region. This improved lower detection limit, at 25-fold over the monomeric scFv on western blot, will assist in detection of biomarkers that are generally low in concentration. Validation through western blot requires the ability to quantify a range of protein concentrations so that healthy, sub-clinical, early stage, and late stage conditions can be monitored. Depending on the biomarker, these abundance ranges can be narrow (fall within 10-fold changes) or wide (span thousand-fold increases) [40]. Sensitivity and a large dynamic range are, therefore, desirable.

We have shown how utilizing mass spectrometry (MS) data, which was collected from a laser-induced retinal injury in a rabbit, can be used to generate recombinant antibodies that if improved further, could aide in the validation of potential biomarkers. By using MS-identified peptides as targets in phage-display library selections of recombinant scFvs, and conversion to the scFv-Fc scaffold, we have increased the chances of recognizing the antigenic epitope existing within the circulating serum. By isolating and enhancing affinity reagents for detecting endogenous proteins by western blot, we have shown that this workflow is an amenable strategy towards biomarker validation. We have also shown how dimerization of the scFv improves the functional affinity through avidity and reduces the lower detection limit in western blotting by 25-fold.

As we have generated recombinant antibodies against peptides, our strategy would be applicable to the immuno-multiple reaction monitoring method of Stable Isotope Standards and Capture by Anti-Peptide Antibodies (SISCAPA) [34]. This technology uses antibodies against peptides to capture candidate biomarkers in a complex sample that have been subjected to proteolytic digestion. The peptide fragments are subsequently enriched for sensitive detection by MS experimentation. In parallel, spiked isotopically labeled versions of the peptides are used as standards to quantify the peptide fragments in the sample. Encouraging evidence of this application was recently reported in the development of high-affinity recombinant Fab antibodies against clinically relevant peptides through phage-display [41]. The Fab antibodies were able to enrich serum spiked peptide fragments and quantify an efficient recovery through comparison to isotopically labeled standards in MS experiments. Although we would require improvement in K_D_ of our scFvs to be useful in these assays [39], the generation strategy is applicable.

## Supporting Information

S1 Fig Amino acid alignment of retinal biomarker scFvs. Clustal W sequence alignment of the two best scFvs from each target peptide. The scFv contains a light chain, followed by a Gly-Ser rich linker and the heavy chain. V_L_ = Variable Light chain. V_H_ = Variable Heavy chain. CDR = Complementarity Determining Region.
(PDF)
