# Supplementary material for: Generating Recombinant Antibodies against Putative Biomarkers of Retinal Injury
Source: PLoS One. 2015 Apr 22;10(4):e0124492. doi: 10.1371/journal.pone.0124492 (PMC4406585; doi:10.1371/journal.pone.0124492)
Supplement: S1 Fig — Clustal W sequence alignment of the two best scFvs from each target peptide. The scFv contains a light chain, followed by a Gly-Ser rich linker and the heavy chain. VL = Variable Light chain. VH = Variable Heavy chain. CDR = Complementarity Determining Region. (PDF) [file pone.0124492.s001.pdf]

```
                                                  VL CDR1                    VL CDR2
GBB5H9        KLNFMLTQPHSVSESPGKAVTISCTGSSGDVARNYVQWYQQRPGSAPIIVIYEDTQRPSG 60
GBB5A1        KLSYELTQPPSVSVSPGQTARITCSG--DALPKKYAYWYQQKSGQAPVLVIYEDSKRPSG 58
RGS9A5        KLQSVLTQPPSASGTPGQRVTSSCSGSRSNIGSNPVAWYQQFPGRAPKLLIYNNNQRPSG 60
RGS9H1        KLSYELTQPPSLSVSPGQTARITCSG--DALPKKYAYWYQQKSGQAPVLVIYEDSKRPSG 58
CNGA3C9       KLNFMLTQPHSVSESPGKTVTISCTGSSGSIANNYVHWYQQRPGSGPTIVIYEDNQRPSG 60
CNGA3A6       KLQAVLTQPRSMSEFPGKTVTISCTGSSGTIASHYVQWYQQRPGSAPATVIYEDNQRPSG 60
CACNA1F2A6    KLQAVLTQPPSVSAAPGQSVAISCSGSSSNIANNYVSWYQLLPGAAPKLLIYDNNKRPSG 60
CACNA1F2A12   KLQSVLTQPPSVSGAPGQRVTISCSGSSSNIGSNSVNWYQQLPGKAPKLLIYYDDLLPSG 60
CACNA1F3A7    KLSYELTQPPSASGTPGQRVTISCSGSSSNIGSNTVNWYQQLPGTAPKLLIYSNNQRPSG 60
CACNA13FA8    KLSYELTQPPSASGTPGQRVTISCSGSSSNIGSNYVYWYQQLPGTAPKLLIYRNNQRPSG 60
              **.  **** * *  **: .  :*:*  . :  : . ***  .* .*  :** :   ***

                                                        VL CDR3
GBB5H9        VPDRFSGSIDSSSNSASLTISGLTTEDEADYYCQSYDG--HNVIFGGGTKLTVLGEGKSS 118
GBB5A1        IPERFSGS--SSGTMATLTISGAQVEDEADYYCYSTDSSGNHGVFGGGTKLTVLGEGKSS 116
RGS9A5        VPDRFSGSK--SGTSASLAISGLQSEDEADYYCAAWDDSLHGWVFGGGTKLTVLGEGKSS 118
RGS9H1        IPERFSGS--SSGTMATLTISGAQVGDEADYYCYSADSSGNP-LFGGGTKLTVLGEGKSS 115
CNGA3C9       VPDRFSGSIDSSSNSASLTISGLKTEDEADYYCQSYNDYGQG-VFGGGTKLTVLGEGKSS 119
CNGA3A6       VPDRFSGSIDSSSNSASLTISGLKTEDEADYYCQSYDS--SSVVFGGGTKLTVLGEGKSS 118
CACNA1F2A6    VPDRFSGSR--SGTSASLAISGLRSEDEADYYCITWDDSLSGPVFGGGTKLTVLGEGKSS 118
CACNA1F2A12   VSNRFSGSK--SGSSASLVISGLQSDDEADYYCATWDDFLNGVVFGGGTKLTVLGEGKSS 118
CACNA1F3A7    VPDRFSGS--KSGTSASLAISGLQSEDEADYYCAAWDDSLNGWVFGGGTKVTVLGEGKSS 118
CACNA13FA8    VPDRFSGSK--SGTAASLAISGLWSEDEADYHCAAWDDSLNGWVFGGGTKLTVLGEGKSS 118
              :.:*****   *.. *:*.***    *****:* : :.     :******:*********

                Linker                                   VH CDR1
GBB5H9        GSGSESKASEVQLVQSGGGLVQPGGSLRLSCAASGFTVSSN--YMSWVRQAPGKGLEWVS 176
GBB5A1        GSGSESKASQVQL-QSGAEVKKPGASVKVSCKASGYTFTGY--YMHWVRQAPGQGLEWMG 173
RGS9A5        GSGSESKASEVQLLESGGGLVQPGGSLRLSCAASGFTFSSY--EMNWVRQAPGKGLEWVS 176
RGS9H1        GSGSESKASEVQLVQSGAEVKKPGSSVKVSCKASGGTFSSY--AISWVRQAPGQGLEWMG 173
CNGA3C9       GSGSESKASEVQLVQSGAEVKKPGSSVKVSCKASGYTFTGY--YMHWVRQAPGQGLEWMG 177
CNGA3A6       GSGSESKASEVQLLESGGGLVQPGGSLRLSCAASGFTFSSY--AMSWVRQAPGKGLEWVS 176
CACNA1F2A6    GSGSESKASEVQLVESGGGLVQPDGSLRLSCTASGFTFTNN--AMSWVRQAPGKGLEWVS 176
CACNA1F2A12   GSGSESKASEVQLVQSGGALVKPGGSLRLSCAASGHSFSDA--WLSWVRQAPGKGLEWVG 176
CACNA1F3A7    GSGSESKASEVQLVQSGAEVKKPGSSVKVSCKASGGTFSSY--AISWVRQAPGQGLEWMG 176
CACNA13FA8    GSGSESKASQVQLQQSGPGLVKPSQTLSLTCAISGDSVSSNSAAWNWIRQSPSRGLEWLG 178
              *********:***  :**  : :*. :: ::*  ** :.:.      *:**:*.:****:.

                   VH CDR2                                            VH CDR3
GBB5H9        SISSSSS--YIYYADSVKGRFTISRDNAKNSLYLQMNSLRAEDTAVYYCA----------- 224
GBB5A1        WINPNSG--GTNYAQKFQGRVTMTRDTSISTAYMELSRLTSDDTAVYYCA----------- 221
RGS9A5        YISSSGS--TIYYADSVKGRFTISRDNAKNSLYLQMNSLRAEDTAVYYCAG---------- 225
RGS9H1        IITPSGG--YTTYAQSFQGRLTMTGDTSTSTVYMELSSLRSEDTAVYYCAY---------- 222
CNGA3C9       WINPNSG--GTNYAQKFQGWVTMTRDTSISTAYMELSRLRSDDTAVYYCARE-------G 228
CNGA3A6       AISGSGG--STYYADSMKGRFTISRDNSKNTLYLQMDSLRVEDTAVYYCANLPGAYCSGA 234
CACNA1F2A6    TIGTSG---DTYYADPVKGRFTISRDNPKNTLYLQMNSLRVEDTALYYCAKN-------- 225
CACNA1F2A12   RIKDKADGETTDYGAPVEGRFTISRDDSKRTLYLQMNSLRTEDTAVYYCTD--------- 227
CACNA1F3A7    GIIPIFG--TANYAQKFQGRVTITADKSTSTAYMELSSLRSEDTAVYYCARGPITMV--R 232
CACNA13FA8    GTYYRSK-WYNDYAVSVKSRITINPDTSKNQFSLQLNSVTPEDTAVYYCARG-------- 229
                           *.  .:. .*:. * .     :::. :  :***:***:

              VH CDR3 cont'd
GBB5H9        ------RSYPFDYWGQGTLVTVSSVDGRAQL--- 249
GBB5A1        ---AGIAVPGFDHWGQGTLVTVSSVDGRAQLM-- 250
RGS9A5        -------GGARDYWGQGTLVTVSSVDGRAQLM-- 250
RGS9H1        --SSGYYFP-FDYWGQGTLVTVSSVDAA------ 247
CNGA3C9       SHSSSWYWDAFDIWGQGTLVTVSSV--------- 253
CNGA3A6       SCSDLYHQHGMDVWGQGTLVTVSSVDGRAQL--- 265
CACNA1F2A6    -----IPGNPFDSWGQGTLVTVSSVDGRAQLMPV 254
CACNA1F2A12   ----------FYYWGQGTLVTVSSVDGRAQLMPV 251
CACNA1F3A7    GDPGYYYYYGMDVWGQGTLVTVSSVDGRAQLMPV 266
CACNA13FA8    ---VLGDSYGMGVWGQGTLVTVSSVDGRAQLMPV 260
                           ***********
```
